# Preparation of Highly Stable and Cost-Efficient Antiviral Materials for Reducing Infections and Avoiding the Transmission of Viruses such as SARS-CoV-2

**DOI:** 10.1021/acsami.3c03357

**Published:** 2023-04-28

**Authors:** Noelia Losada-Garcia, Angela Vazquez-Calvo, Antonio Alcami, Jose M. Palomo

**Affiliations:** † Instituto de Catálisis y Petroleoquímica (ICP), CSIC, C/ Marie Curie 2, 28049 Madrid, Spain; ‡ Centro de Biología Molecular Severo Ochoa, Consejo Superior de Investigaciones Científicas (CSIC)−Universidad Autónoma de Madrid (UAM), 28049 Madrid, Spain

**Keywords:** antiviral material, SARS-CoV-2, copper, viruses, surface coating

## Abstract

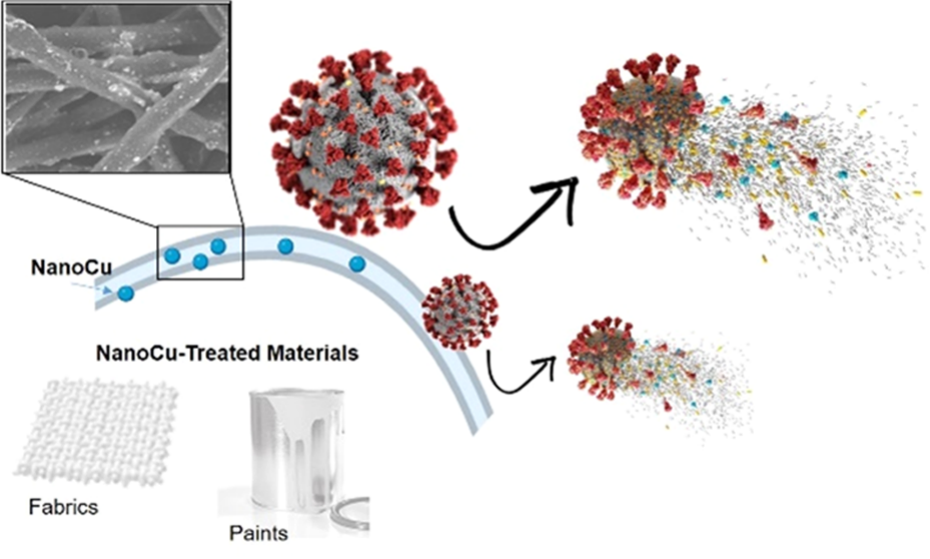

The current global pandemic due to the severe acute respiratory syndrome coronavirus 2 (SARS-CoV-2) virus has demonstrated the necessity to develop novel materials with antimicrobial and antiviral activities to prevent the infection. One significant route for the spread of diseases is by the transmission of the virus through contact with contaminated surfaces. Antiviral surface treatments can help to reduce or even avoid these hazards. In particular, the development of active-virucidal fabrics or paints represents a very important challenge with multiple applications in hospitals, public transports, or schools. Modern, cutting-edge methods for creating antiviral surface coatings use either materials with a metal base or sophisticated synthetic polymers. Even if these methods are effective, they will still face significant obstacles in terms of large-scale applicability. Here, we describe the preparation of fabrics and paints treated with a scaled-up novel nanostructured biohybrid material composed of very small crystalline phosphate copper(II) nanoparticles, synthesized based on a technology that employs the use of a small amount of biological agent for its formation at room temperature in aqueous media. We demonstrate the efficient inactivation of the human coronavirus 229E (HCoV-229E), the severe acute respiratory syndrome coronavirus 2 (SARS-CoV-2) virus, and non-enveloped human rhinovirus 14 (HRV-14) (>99.9%) using an inexpensive, ecologically friendly coating agent. The reactive oxygen species produced during the oxidation of water or the more intensive reaction with hydrogen peroxide are believed to be the cause of the antiviral mechanism of the nanostructured material. In contrast to the release of a specific antiviral drug, this process does not consume the surface coating and does not need regeneration. A 12-month aging research that revealed no decline in antiviral activity is proof that the coating is durable in ambient circumstances. Also, the coated fabric can be reused after different washing cycles, even at moderate to high temperatures.

## Introduction

Worldwide, respiratory pathogens are the leading cause of infectious mortality.^1,2^ The current global COVID-19 pandemic caused by the severe acute respiratory syndrome coronavirus 2 (SARS-CoV-2) infection^3^ has generated a catastrophic scenario both economically and socially, with almost 6.9 million deaths and more than 750 million infected people worldwide.^4^ The transfer of viruses through contact with contaminated surfaces is an important pathway for the spread of infections. This has demonstrated the necessity to develop novel materials with antimicrobial and antiviral activities to prevent the infection of actual viruses and others that could appear in the near future.^5−9^

Antiviral surface material treatments can have two effects: reduce or directly avoid these hazards. Self-cleaning materials in which virus particles are directly inactivated when they are in contact with the material is highly desired.^10,11^

In particular, the development of active-virucidal fabrics or paints represents a very important challenge regarding their multiple applications in hospitals, public transports, or schools. Modern, cutting-edge methods for creating antiviral surface coatings either use materials with a metal base or sophisticated synthetic polymers.^5−9,12−15^ Even if these methods are effective, they will still face significant obstacles in terms of large-scale applicability and environmental sustainability.

Among the different metals, copper represents a good alternative in destroying microbial and viral pathogens, with more efficiency than zinc and less toxicity than silver.^16,17^

In this regard, different technologies have been developed in the preparation of copper compounds as coating agents, for example, in the fabrication of face masks or antiviral textiles.^18−21^ Especially in the last 2 years, different products containing copper materials have been commercialized, mainly using a mixture of species such as Cu_2_O or CuO, or directly using the bulk copper.^22−24^ This poses several questions because the amount of copper is extremely high and could have important toxicity problems. Also, some of these species need to be provide copper ion release to be effective. Although it is well known that copper ions may effectively inactivate viruses by targeting their nucleotide sequences, a different strategy must be used when a virus particle—as a whole—needs to be destroyed. Although the inactivation of viruses appears to be caused by the production of reactive oxygen species (ROS) (Figure S1), the presence of oxidative substances like peroxide tends to enhance copper’s biocidal effect. For instance, in the case of enveloped viruses, a first attack on the membrane is required. Then, a variety of inhibition processes can be carried out by the copper, inhibiting virus proteases and inactivating viral metalloproteins, binding to their nucleotides, cross-linking the strands, or fragmenting them, which cannot be repaired because there are no nucleic acid repair mechanisms.^25^

In terms of efficiency, the application of nanotechnology represents a new alternative. If the virus inactivation is considered a catalytic oxidative process, the use of nanocatalysts seems to be an advantage compared to bulk material, considering the intrinsic properties of nanoparticles—with an extremely large surface-to-volume ratio. In terms of applicability, durability of the coating agent on the material is essential, conserving the antiviral capacity after washing in the case of textiles, or a mechanism that does not need regeneration of the material, for example, in paint application.

**Figure 1:**
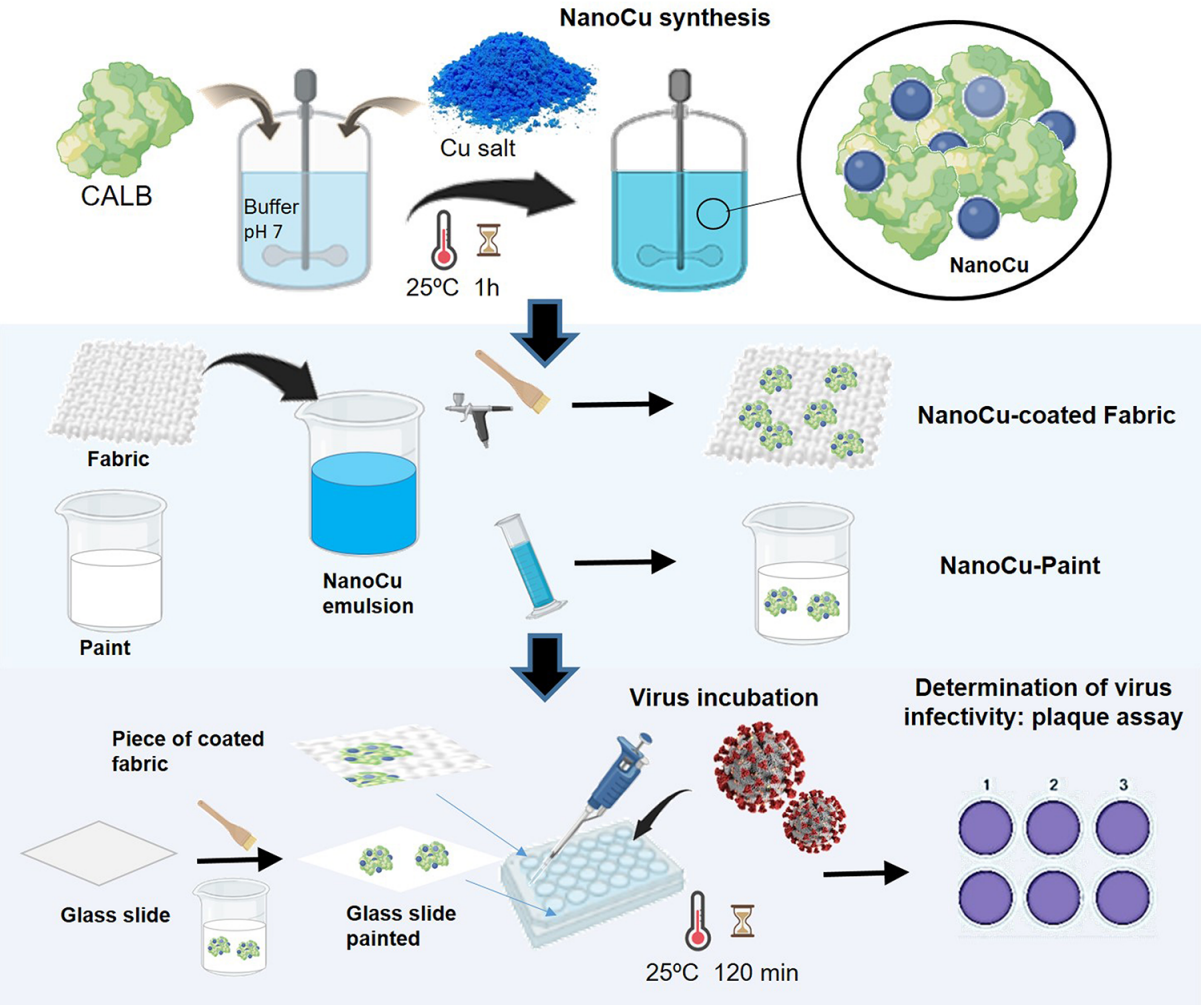
General concept of the fabrication of antiviral NanoCu-coated materials.

In this work, we demonstrate how to fabricate a new type of coated antiviral fabrics or paints considering both aspects. For this, we synthesized a coating agent as a liquid emulsion based on a nanostructured biohybrid material, which is composed of very small copper(II) nanoparticles (as active sites) embedded in a protein network in aqueous media. The (lipase from *Candida antarctica* B) enzyme used (as a scaffold) is a robust and cheap commercial available enzyme supplied by Novozymes. This enzyme is applied directly without any treatment in the fabrication of this nano-biohybrid and it plays a key role in the *in situ* formation and stabilization of the nanoparticles in the protein network, maintaining their homogeneous distribution and avoiding nanoparticle aggregation. Also, the protein structure is important in the final physico-chemical interaction with the coated materials (based on its multiple hydrophobicity and the ionic nature of the amino acid residues in its structure) (Figures 1 and S2).

The synthesis is performed in a very sustainable way, in aqueous media and room *T*, and it demonstrated the easy scale-up of the process, a critical step for the final potential commercialization of the product.

This copper-protein material was added as a coated agent in fabrics with different compositions and as a typical paint by using different technologies, and sprayed or immersion processes. The stability of the coated materials was studied in the presence of different domestic agents (bleach, hydrogen peroxide, detergents), at different temperatures or even time aging at ambient conditions.

The coating agent showed excellent virucidal activity against human coronavirus 229E (HCoV-229E), the severe acute respiratory syndrome coronavirus 2 (SARS-CoV-2) virus, and also a non-enveloped human rhinovirus 14 (HRV-14), extending the applicability of the method by an action mechanism following the description above (Figure S1). Reusability experiments of the coated materials were performed and antiviral activity was found to be conserved.

## Materials and Methods

### Materials

Copper(II) sulfate pentahydrate [Cu_2_SO_4_·5H_2_O], hydrogen peroxide (H_2_O_2_, 33%), and sodium hydroxide (NaOH) were obtained from Panreac (Barcelona, Spain). Lipase from *C. antarctica* (CALB) solution was obtained from Novozymes (Copenhagen, Denmark). Hexadecyltrimethylammonium bromide (CTAB) was obtained from Affymetrix (California, EE.UU). 0.6% (v/v) didecyldimethylammonium chloride (C_22_H_48_ClN) (commercial product Multipurpose Green Forest Disinfectant) and bleach (obtained by diluting commercial bleach (40 g sodium hypochlorite/L) were obtained from a commercial store (Madrid, Spain). Luria–Bertani (LB) medium was purchased from Formedium (Norfolk, England). Syrris/Orb Jacked Reactor was purchased from M.T. BRANDAO ESPAÑA, S.L. (Tres Cantos, Spain). Vero-E6, HuH-7 cells (kindly provided by Isabel Solá and Luis Enjuanes, CNB-CSIC, Madrid, Spain), and HeLa-H1 (kindly provided by Mauricio García-Mateu, CBMSO, Madrid, Spain) were grown in Dulbecco’s modified Eagle’s medium (DMEM; Invitrogen) containing 5% fetal bovine serum (FBS), 2 mM l-glutaminie, 100 mg/mL streptomycin, and 100 U/mL penicillin, and incubated at 37 °C/5%CO_2_. The viruses used were human coronavirus 229E (HCoV-229E), severe acute respiratory syndrome coronavirus 2 (SARS-CoV-2) strains MAD6 (kindly provided by Isabel Solá and Luis Enjuanes, CNB-CSIC, Madrid, Spain) and human rhinovirus 14 (HRV-14) (kindly provided by Mauricio García-Mateu, CBMSO, Madrid, Spain). All infectious virus manipulations were performed in our biosafety level 2 (BSL-2) or BSL-3 facilities. The fabrics were supplied by the company HIGITEX (Spain). The paint was provided by AC Pinturas (Vélez-Málaga, Spain). The MC360 equipment was lent by Microclean-solutions (Madrid, Spain).

### Characterization Methods

Scanning electron microscopy (SEM) imaging was performed using a TM-1000 microscope (Hitachi, Tokyo, Japan) by adding a small amount of the sample to a thin film with a layer of conducting carbon. Transmission electron microscopy (TEM) and high-resolution TEM microscopy (HRTEM) images were obtained using a TEM/STEM microscope (JEOL 2100F, Tokyo, Japan) operating at 200 kV with a field emission filament, yielding a theoretical point resolution of 0.19 nm, equipped with an EDX INCA x-Max80 detector (Oxford Instruments, Abingdon, U.K.) that allows the possibility of performing semiquantitative chemical analyses. Samples for TEM analysis were prepared by placing a drop of sample solution on a copper grid coated with an amorphous carbon film. Interplanar spacing in the nanostructures was calculated using the inversed Fourier transform with the GATAN digital micrograph program (Corporate Headquarters, Pleasanton, CA). X-ray diffraction (XRD) patterns were obtained using a PANalytical X’Pert Pro polycrystalline X-ray diffractometer with a D8 Advance texture analysis Θ-Θ setup (Bruker, Billerica, MA) with Cu Kα radiation (λ = 1.5406 Å, 45 kV, 40 mA). Their analysis was performed using the X’Pert Data Viewer and X’Pert Highscore Plus programs. X-ray photoelectron spectroscopy (XPS) spectra were determined using a SPECS GmbH electronic spectroscopy system with an ultrahigh vacuum (UHV) system (pressure approximately 10–10 mbar), with a PHOIBOS 150 9MCD energy analyzer and monochromatic X-ray sources. The analysis of the same was carried out using the CasaXPS program. Inductively coupled plasma-optical emission spectroscopy (ICP-OES) was performed of the solid material. 10 mg of the solid powder was treated with 5 mL of HCl (37% v/v) for digestion. Then, it was added with 5 mL of water, centrifuged, and the clear solution was analyzed for Cu content. Inductively coupled plasma-optical emission spectrometry (ICP-OES) was performed using an OPTIMA 2100 DV instrument (PerkinElmer, Waltham, MA).

### Synthesis of NanoCu

18 mL of commercial CALB solution (containing 9 mg lipase/mL) was added to 600 mL of sodium phosphate buffer, 0.1 M, pH 7, in a 1 L glass bottle containing a small magnetic bar stirrer or in an open/closed glass with stirring blade propellers at room temperature. Then, 6 g of Cu_2_SO_4_·5H_2_O (10 mg/mL) was added to the protein solution and the mixture was incubated for 16 h. After the first 30 min of incubation, the solution turned cloudy (turquoise). After this time, an emulsion with a concentration of 5000 ppm, the so-called NanoCu, was obtained. Characterization of the Cu hybrid was performed by XRD, SEM, TEM, HRTEM, IR, and XPS.

### Optimization and Scale-Up Preparation of NanoCu

**Figure 2:**
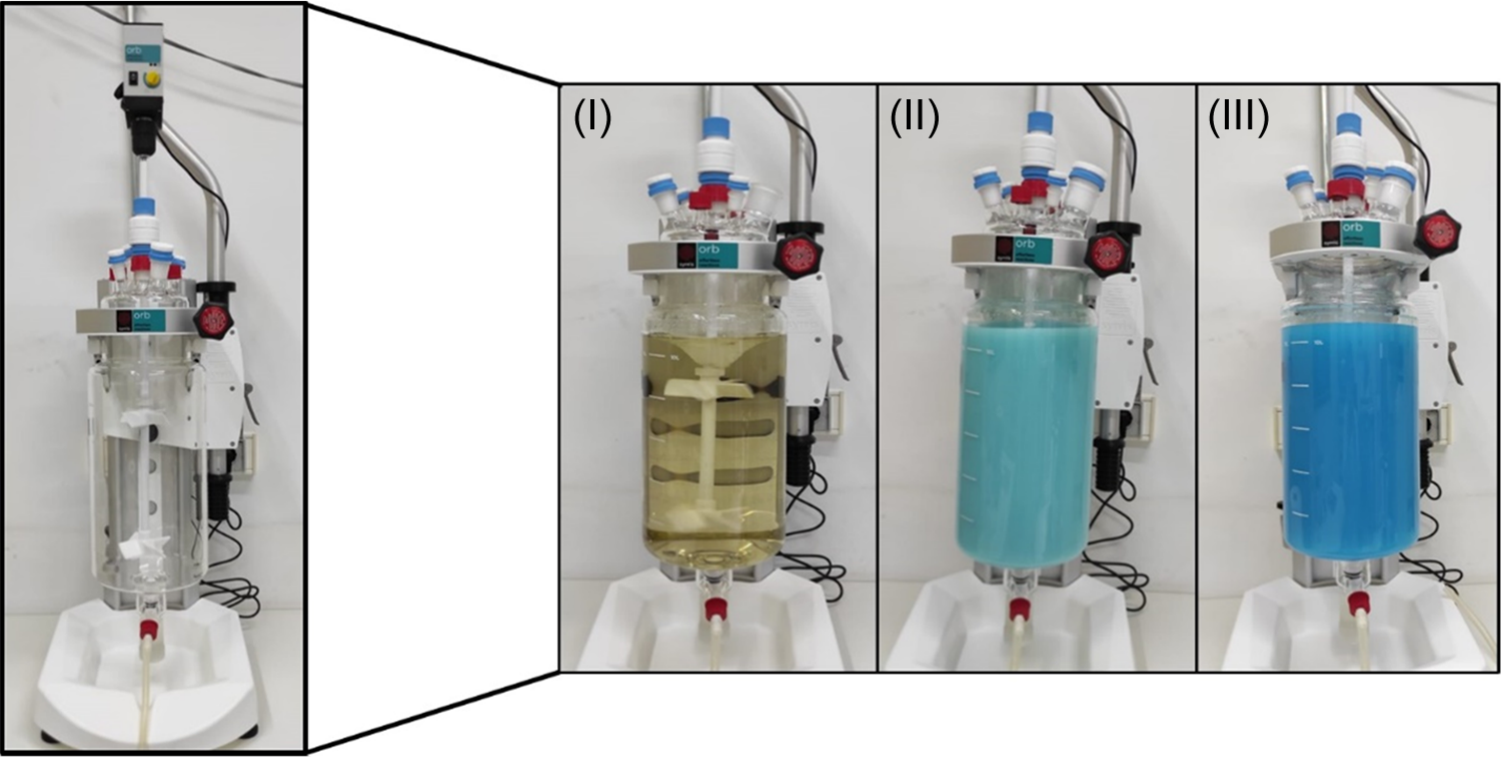
NanoCu scaling process in the 10 L reactor. (I) CALB addition *t*(0); (II) synthesis at 15 min; (III) synthesis at 1 h.

The reaction described above was repeated at different conditions, by stopping at different incubation times (8, 4, or 1 h) or evaluating from 20 to 40 °C. The synthesis was scaled up to 3, 5, and 10 L in a Syrris/Orb Jacked Reactor couple with a paddle stirrer as shown in Figure 2 for 1 h incubation. For the 10 L synthesis, 156 g of sodium phosphate was first added to 10 L of distilled water using a stirrer. Then, 200 mL of 3 M NaOH was added to adjust the pH at 7.00. Then, 267 mL of commercial CALB was added, followed by 100 g of CuSO_4_·5H_2_O. The mixture was incubated for 1 h and then emulsion was directly collected in a 25 L drum, obtaining NanoCu. A second approach was to add 125 mL 0.5% (v/v) of hydrogen peroxide (33.3%) to 25 L of NanoCu to obtain the emulsion NanoCu/H_2_O_2_ as a NanoCu solution.

### Model Catalytic Oxidation Reaction to Determine NanoCu Activity

*p*-Aminophenol (*p*AP) (1 mg) was dissolved (10 mL) in distilled water and 100 mM hydrogen peroxide (1%, v/v) was added. To initiate the reaction, 10 mL of this solution was added to a glass bottle containing 130 μL of 500 ppm solution (3 mg) in a NanoCu or Petri dish or a small crystallizer containing a piece of coated fabric (10.5 cm^2^) and stirred gently at room temperature on an orbital shaker (320 rpm). At different times, supernatant samples (100 μL) were taken and the reaction was followed by high-performance liquid chromatography (HPLC). Samples were first centrifuged at 8000 rpm for 5 min and then 50 μL was diluted 20 times in bi-distilled water before injection. The HPLC column was C8 Kromasil 150 × 4.6 mm^2^ AV-2059. HPLC conditions were an isocratic mixture of 15% acetonitrile and 85% bi-distilled water, UV detection at 270 nm, and a flow rate of 0.4 mL/min. The possible adsorption of substrate to the catalyst was first tested; without the presence of hydrogen peroxide no reaction was observed, and the full area of the substrate was unaltered in the HPLC analysis.

### Coating of Polypropylene Fabrics with NanoCu

5000 ppm NanoCu solution was diluted up to 1000 ppm to obtain mixtures of ethanol/water at different concentrations (50:50, 70:30, and 80:20). Using a brush, the surface of 45 cm^2^ of a polypropylene fabric (e.g., face mask) was homogeneously covered with subsequent drying by direct heating between 30 s and 2 min. For ethanol/water 50:50, different samples (45 cm^2^) were prepared by applying different amounts of NanoCu (1000, 750, 500, 250, 125 ppm) and characterized.

### Coating of Polyester Fabrics with NanoCu

Polyester fabrics were coated with NanoCu solution by using a brush or by the dipping process in a NanoCu-diluted aqueous solution. In the first approach, a similar protocol was followed as described above, starting with NanoCu in ethanol/water 50:50 at 125–350 ppm concentration. In the second approach (a more industrial process), NanoCu solution (5000 ppm) was diluted with distilled water at 625 ppm (*a*) or 1250 ppm (*b*) and added to a glass bath. A piece of fabric of 10.5 cm^2^ was dipped in the bath with agitation (magnetic stirrer) in case *a* and without agitation in case *b*. The immersion process was carried out for 2 min in case *a* and for 1 min in case *b*. Then the pieces were recovered and dried with hot air (around 60 °C) for 30 s. The coated fabrics were characterized by electron microscopy (SEM).

### Coating of Fabrics with NanoCu by Spraying Methods

Another strategy tested to coat the fabrics (polypropylene, 100% cotton, polyester (black and white)) was the use of the spray method (electrostatic or mechanical). The electrostatic spray method was performed using MC360 equipment. NanoCu was applied in a single concentration, 5000 ppm, through a flow rate of 118 mL/min at a distance of about 1 m. The spraying process was carried out for different times (1–10 s) depending on the material. Subsequently, each coated fabric was subjected to a drying process with hot air for 30 s. In the case of manual spray using a sprayer, two different concentrations of NanoCu, 1250 and 625 ppm (diluted with water starting from a 5000 ppm NanoCu solution), were used. The spraying process was carried out by combining different positions and sprays: specifically, spray nine times at 5 cm or 15 cm horizontally, at 45° inclination; spray 15 times at 5–10 cm, or 12 times at 20 cm, from the vertical position. Then, each coated fabric was subjected to a drying process with hot air for 30 s each.

### Experiments of Washing Cycles of the Coated Fabrics

NanoCu-coated fabrics (45 cm^2^ containing 125 ppm) were washed using different protocols: (i) two washing steps with tap water, one at 40 °C and another at 60 °C for 2 h, to check their structural stability; (ii) series of three washes at different times (30 min, 1 h, and 2 h) with tap water at 40 and 60 °C; (iii) a water bath was prepared at 25 °C and consecutive washes (six cycles) were performed for 30 min each. After the last wash step in each case, the piece of fabric was left to air-dry overnight. ICP-OES analysis was performed to determine the amount of copper retained and for SEM analysis.

### NanoCu-Paint Mixed Preparation

NanoCu was directly added to 100 mL of paint as additive from a 5000 ppm solution at different concentrations between 5 and 50% (v/v) and mixed in a paddle stirrer for 10–15 min. Then, NanoCu paint was homogeneously distributed (10 mL) in a Petri dish and was dried at 37 °C for 2–4 h. Then, the oxidative catalytic activity of NanoCu, XRD analysis, and virucidal analysis were performed on the dish.

### Antiviral Tests against Different Viruses of NanoCu-Coated Fabrics or Paint

The virus yield was determined by plaque assay in HuH-7 cells (HCoV-229E), Vero-E6 cells (SARS-CoV-2), or HeLa-H1 (HRV-14). Cells were seeded into 12-well plates and incubated at 37 °C for 24 or 48 h (in case of HuH-7) in 5% CO_2_. Then, the cells were inoculated with 0.2 mL of the serial 10-fold diluted harvested supernatant. For HCoV-229E, after 2 or 1 h of absorption at 37 °C the virus inoculum was removed; the medium containing 0.7% agarose, diethylaminoethyl (DEAE)-dextran (0.090 mg/mL), and 2% FBS was added; and the plates were incubated for 4 days at 33 °C. For SARS-CoV-2, after inoculum removal, infections were allowed to proceed in a semisolid medium containing 1.5% carboxymethyl cellulose, 10 mM *N*-(2-hydroxyethyl)piperazine-*N*′-ethanesulfonic acid (HEPES), and 2% FCS, and the plates were incubated at 37 °C for 3 days. In the case of HRV-14, after inoculum removal, the medium containing 0.7% agarose, DEAE-dextran (0.045 mg/mL), and 2% FBS was added, and the plates were incubated for 3 days at 35 °C. At this time point, cells were fixed in 4 or 10% formaldehyde for at least 30 min at room temperature and stained with 3% crystal violet in 2% formaldehyde. Finally, the plates were washed and the viral plaques were counted.

For virucidal assay, fabrics coated or uncoated as described above were cut into 1 × 1 cm^2^ squares. 50 μL of viral stock containing ∼5 × 10^5^ PFU of HCoV-229E, SARS-CoV-2, or HRV-14 were added to the center of each sample and incubated at room temperature for 2 h. Then, each sample was washed with 950 μL of DMEM 2% FBS and the recovery virus titer was determined by plaque assay as described previously. In the case of paint, 12 mm coverslips were painted with 25 μL of water paint mixed with 50% NanoCu solution or water paint mixed with 50% deionized water + 0.5% H_2_O_2_ as control and air-dried overnight. Then, 50 μL of viral stock containing ∼5 × 10^5^ PFU of HCoV-229E or SARS-CoV-2 was added to a coverslip and incubated at room temperature for 0, 15, or 30 min at room temperature or for 2 h at 4 °C. Then, each sample was washed with 950 μL of DMEM 2% FBS and the recovery virus titer was determined by plaque assay as described previously.

## Results and Discussion

### Synthesis, Characterization, and Optimization of NanoCu

**Figure 3:**
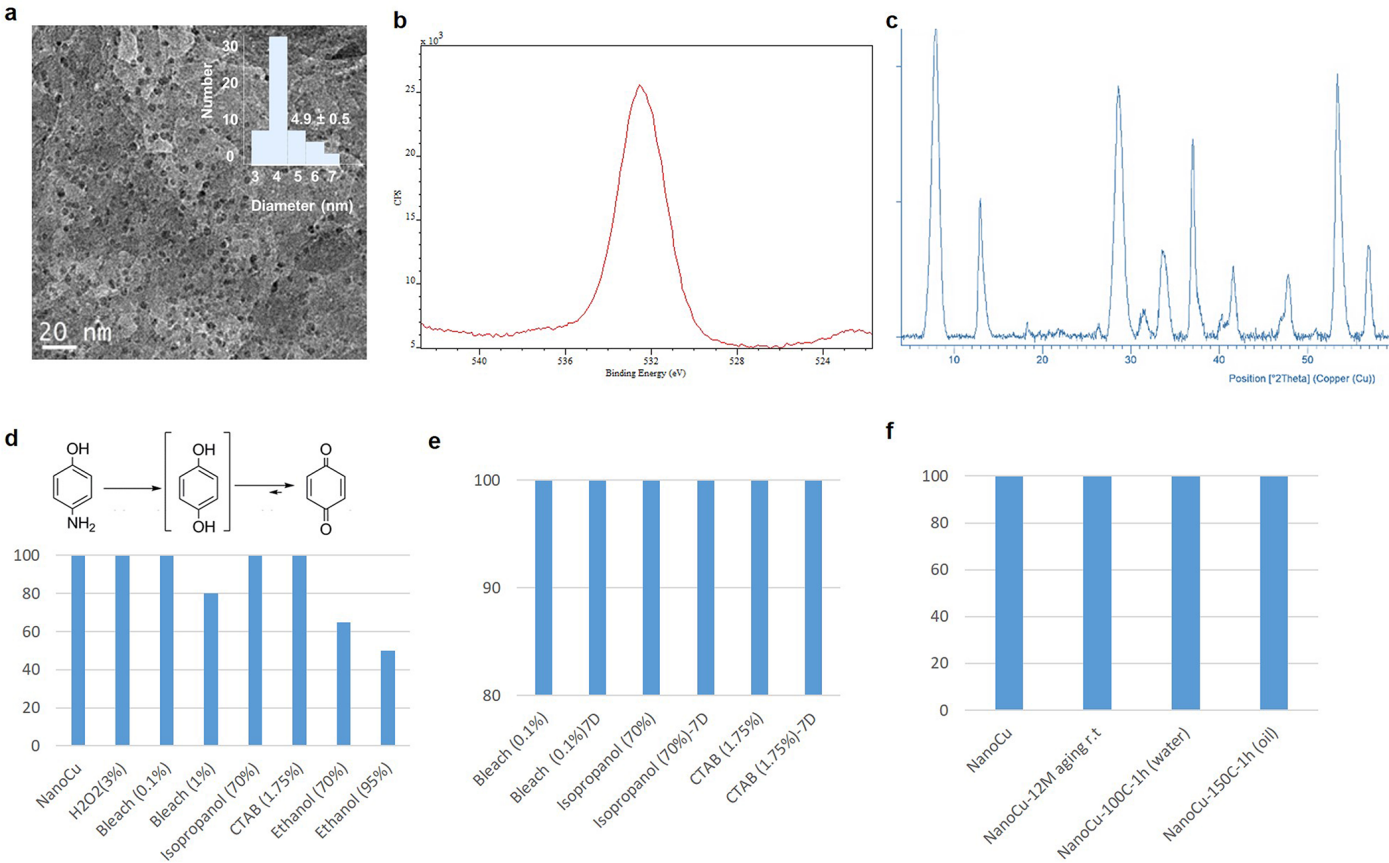
Characterization and stability evaluation of NanoCu. (a) HRTEM image (inset particle size distribution). (b) XPS spectrum of O 1s. (c) X-ray diffraction pattern. (d–f) Fenton catalysis in the hydroxylation reaction of *p*-aminophenol to benzoquinone. NanoCu after 5 min reaction involving full conversion (100% activity as control) and activity after adding different additives or different conditions.

The copper nanostructured biohybrid material (NanoCu) was synthesized in a mild condition, room temperature and neutral pH medium. The process is based on a bio-induced metal nanoparticle formation, where commercial available CALB (2.9%, v/v) is mixture together with copper salt (1%, w/v) in phosphate buffer solution (Figure 1). First batch was made at 600 mL amount and 16 h incubation time. Characterization of the blue emulsion formed showed the formation of nanoflower structures (data not shown) containing very small crystalline spherical nanoparticles of around 4 nm diameter size (TEM analysis) (Figure 3a). Cu species in NanoCu was exclusively copper(II) phosphate, confirmed by XPS analysis (Figure 3b) and XRD (matched well with JCPDS card no. 01-080-0991)^26^ (Figure 3c).

For potential industrial application, stability of the copper species in NanoCu was evaluated at different conditions, high temperatures, or the presence of different additives, mainly with disinfectant properties. For that, catalytic activity of NanoCu in the oxidation of p-aminophenol to benzoquinone in the presence of hydrogen peroxide was used as model reaction (Figure 3d–f). The initial catalytic activity of NanoCu in *p*AP oxidation was fully conserved in all cases, in the presence of isopropanol (70%), hydrogen peroxide (3%), bleach (0.1%), or CTAB (1.75%) and more than 80% in the presence of 1% Bleach. Indeed, these results were conserved after 7 days of incubation (Figure 3f). NanoCu structure was conserved confirmed by XRD (Figure S3).

Although the fabrication of NanoCu is a green and sustainable process, scaling studies are mandatory. Therefore, the synthetic protocol was optimized from 16 to 1 h and scaled up from 0.6 to 10 L in a Syring reactor (Figure 2). Reproducibility in the synthesis was confirmed after 30 cycles obtaining identical data of XRD pattern (Figure S4), TEM, ICP-OES, and catalytic assay in all batches. This confirmed that synthesis can be carried out in aqueous media at room temperature without the need for special conditions or special equipment.

### Fabrication of NanoCu-Coated Fabrics

**Figure 4:**
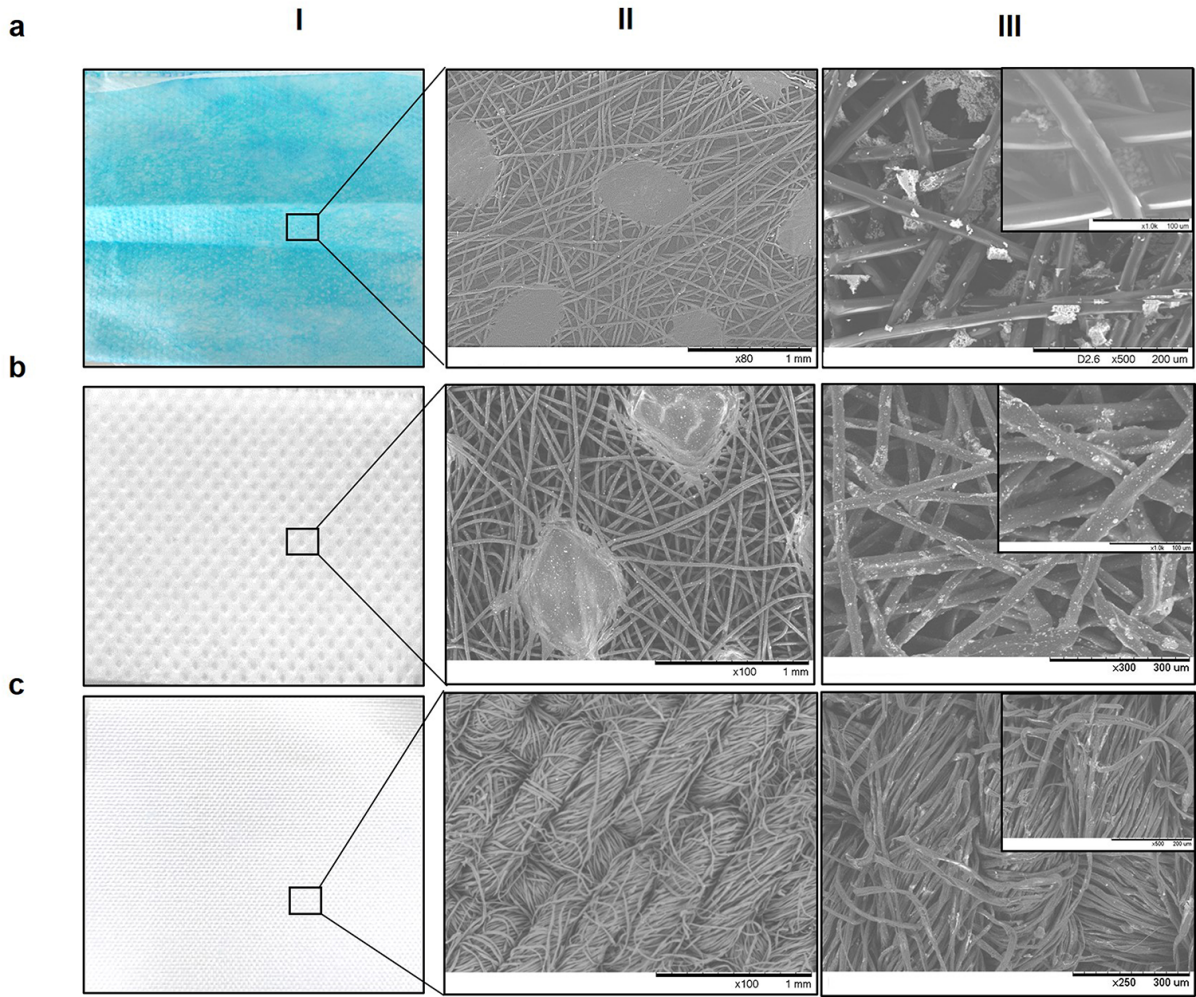
NanoCu coating on different surfaces. (a) Polypropylene fabric with 125 ppm on 45 cm^2^. (b) Filter fabric with 125 ppm on 45 cm^2^. (c) Cotton-fabric mask with 125 ppm for 45 cm^2^. (I) Part of the coated fabrics. (II) SEM images of the uncoated fabric. (III) SEM images of the coated fabric with NanoCu.

NanoCu was applied directly as a uniform coating on the surface of a commercial polypropylene fabric (non-woven, surgical mask piece) (Figure 4a). NanoCu was homogeneously incorporated into the surface of the first layer of a piece of facial mask of 45 cm^2^ (1/4 of complete mask) by a solution method, where different amounts of NanoCu (125–1000 ppm) (Figures S5 and S6) were applied. Scanning electron microscopy (SEM) images of the coated fabric verified the presence of the material adhered to the fibers of the fabric (Figure 4a- III), with an optimized amount of 125 ppm. This could be attributed to the protein network existing in NanoCu, which can bind to the fabric fiber being embedded on the material and not supported on the surface. Importantly, under these conditions, the amount of material did not obstruct the channels necessary for the breathability of the mask (Figure 4). Also, the coated mask was treated in the presence of ethanol (from 0 to 100%) and further washed with water at 50 °C and no leaching of NanoCu was observed in any case, confirmed by ICP-OES mass analysis (data not shown). NanoCu was subsequently applied to another type of fabric, similar material used for example in FFP2 mask, a mixture of polypropylene and polyethylene (Figure 4b). SEM images showed the NanoCu was perfectly adhered to fabric mainly into the fibers (Figure 4b). On the other hand, using fabrics as cotton (100%) (Figure 4c) or polystyrene (Figure S7) similar results were found.

**Figure 5:**
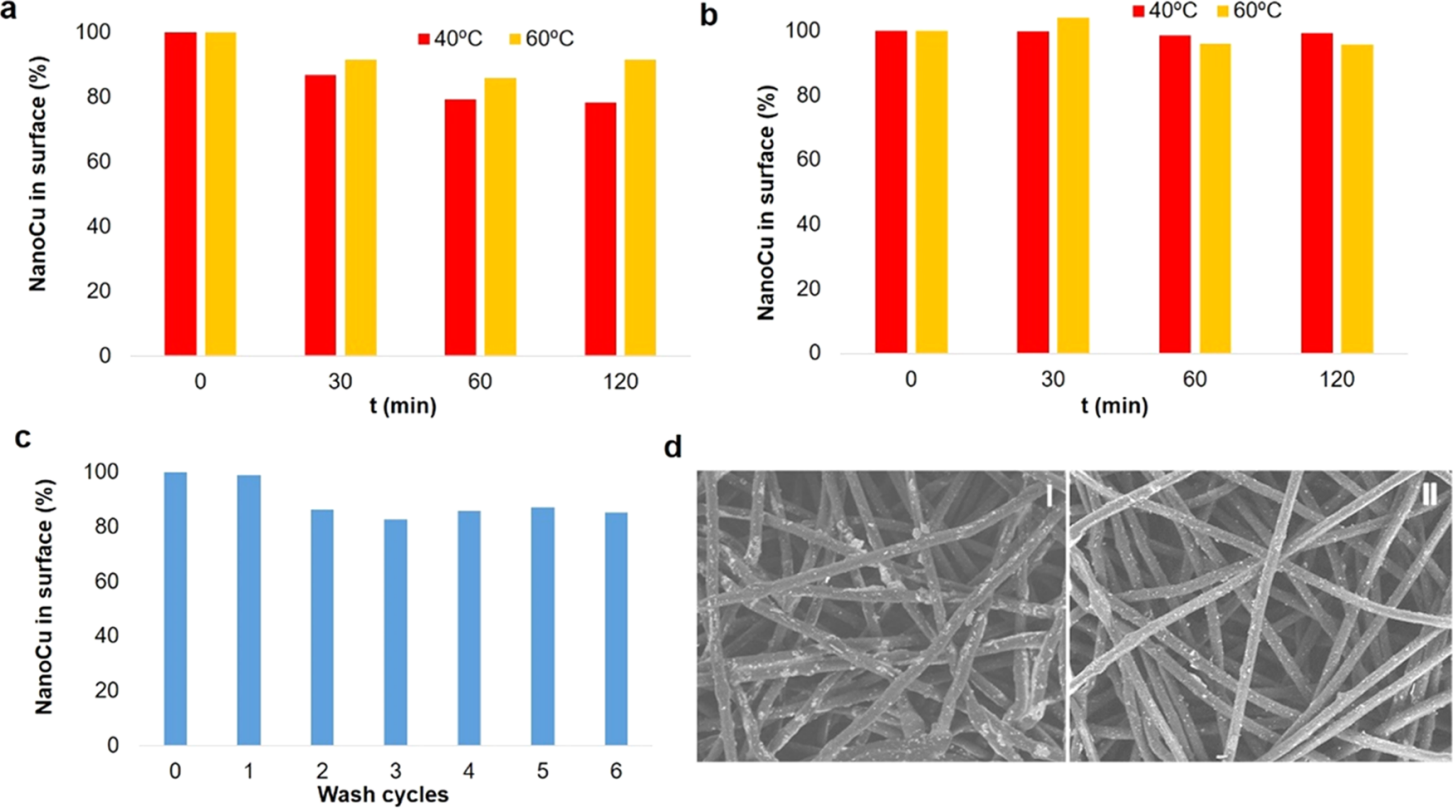
(a) Reusability and maintenance of NanoCu in the polypropylene fabric at 40 and 60 °C for different times (0–120 min). (b) Reusability and maintenance of NanoCu in the cotton-fabric mask at 40 and 60 °C for different times (0–120 min). (c) Reusability and maintenance of NanoCu in the fabric at 25 °C during six washes. (d) SEM images (300 μm scale) of the fabric coated with NanoCu: (I) initially (no washes); (II) after six wash cycles.

Afterwards, the different NanoCu-coated fabrics were evaluated in terms of potential reuse. For this, two different tests were carried out. First, a piece of polypropylene fabric (45 cm^2^) and a 10.5 cm^2^ cotton fabric were incubated, respectively, at 40 °C and 60 °C for 30, 60 and 120 min (Figures 5a,b and S8).

Second, a polypropylene/polyethylene (filter) fabric was evaluated at 25 °C for six washing cycles (Figure 5c,d). In all cases, between 85 and 100% of NanoCu was maintained on the surface, confirmed by ICP-OES and SEM analyses.

Other materials such as filter polystyrene or polyester fabrics by using immersion or spraying strategies resulted also in a homogeneous distribution of NanoCu (Figures S9–S11).

The efficient activity of NanoCu-coated fabrics was tested using the previous model assay of oxidation of *p*AP, yielding in a similar result than using NanoCu in emulsion form (data not shown).

### Virucidal Activity of NanoCu-Coated Fabrics

The antiviral efficiency of the NanoCu-coated fabrics (cotton, fiber or white (polyester)) was first tested against HCoV-229E coronavirus in biosafety level 2 (BSL-2) lab (Figure S12). The final efficiency depended on the material, and fiber or white coated with NanoCu showed 90% inhibition whereas cotton-NanoCu showed 99% inhibition. No inhibition was observed with the uncoated fabrics (Figure S12). Also the free enzyme was tested demonstrating that only copper nanoparticles were responsible of antiviral activity (data not shown). Preliminary results with NanoCu in solution showed better performance in the presence of a small amount of hydrogen peroxide (0.16%) (data not shown). Thus, different coated fabrics were prepared using NanoCu which hydrogen peroxide was added to the emulsion, employing this new emulsion NanoCu* in the same way as previously described.

**Figure 6:**
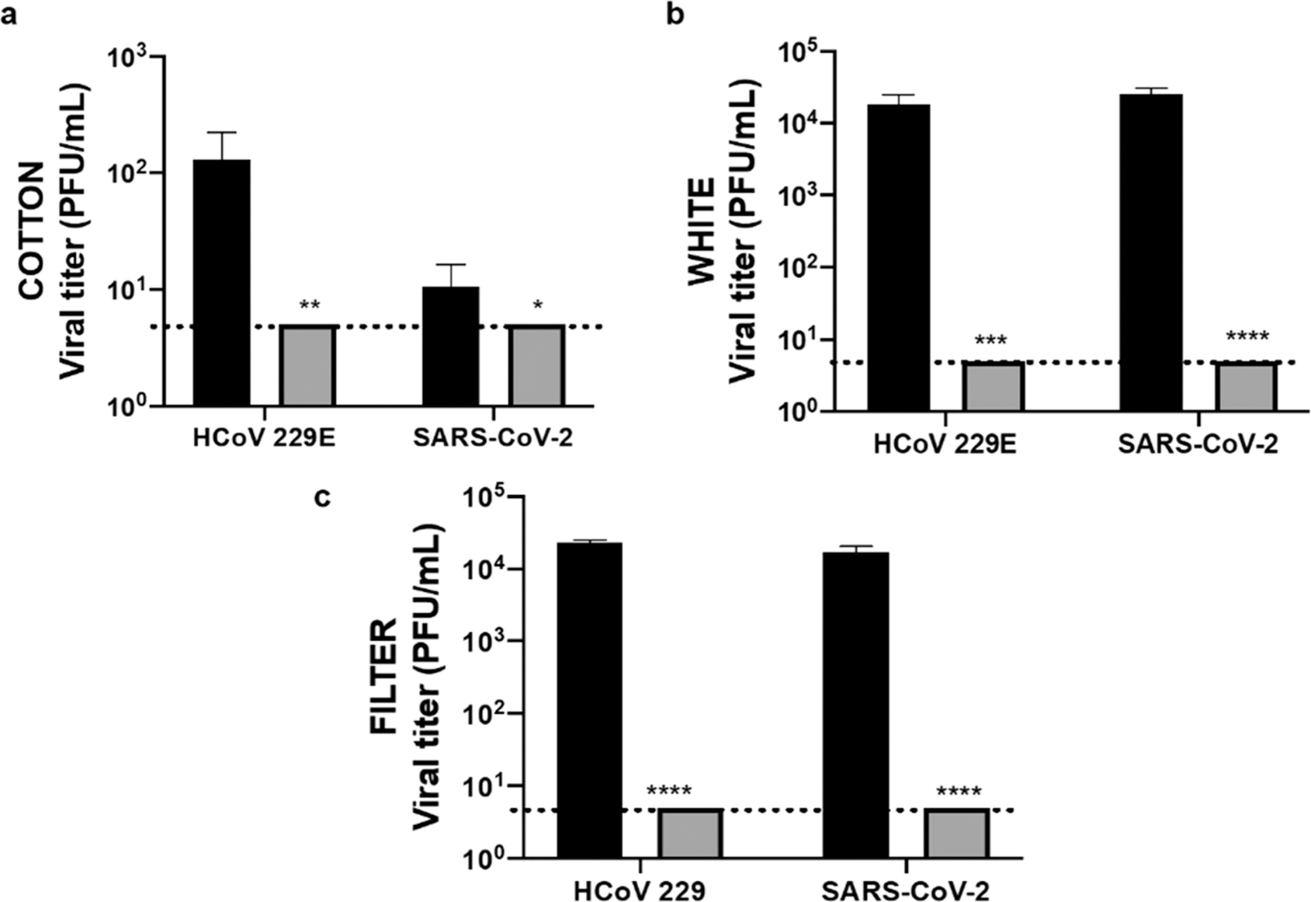
Determination of the virucidal activity of different fabrics with NanoCu* incorporated against coronavirus. A volume of 50 μL of viral inoculum containing approximately 10^5^ PFU of HCoV-229E or SARS-CoV-2 virus was applied to different textiles and incubated for 120 min at room temperature. Then, the recovery virus titer was determined by plaque assay. Dotted lines represent the limit of detection by the assay. (a) Cotton fabric. (b) White polystyrene fabric. (c) Filter fabric. *N* = 4 replicates were done, and the errors bars correspond to ±standard deviation (±SD). Paired Student test analysis was employed for comparing the experimental treatments with the control: *p < 0.05; **p < 0.01; ***p < 0.005; ****p < 0.001. Control without fabric (black bars), NanoCu*-coated fabric (gray bars).

Then, virucidal activity of this coated fabric was tested against HCoV-229E and SARS-CoV-2 virus in biosafety level 3 (BSL-3) lab (Figure 6). In the inhibition of HCoV-229E, cotton-coated fabric showed similar results using NanoCu* than NanoCu, with a reduction in the number of viruses of 2 log 10. However, in the case of fiber or white fabrics, the coating with NanoCu* showed a more virucidal textile, with more than 4 log 10 of virus reduction (99.99% efficiency) in both cases (Figure 6b,c). These excellent results (99.99% efficiency) of virus inhibition efficiency were also obtained against SARS-CoV-2 (Figure 6b,c).

The virucidal capacity of these coated fabrics was in addition tested against a non-enveloped virus such as human rhinovirus (HRV-14) (Figure S13). In this case, once more the coating of fabrics such as filter (polypropylene/polyethylene) or white (polyester) produced antiviral fabrics against this virus with 99.9% inhibition, demonstrating the versatility of this coating process.

**Figure 7:**
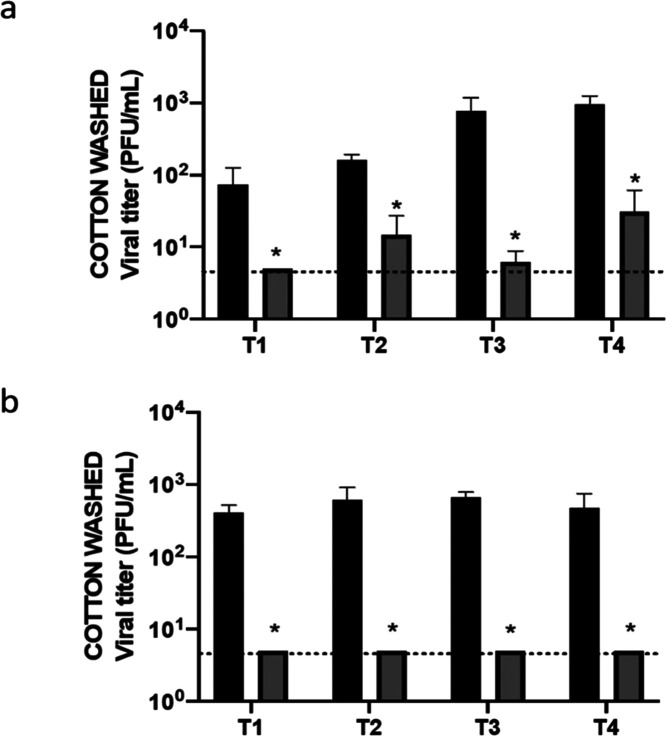
Determination of virucidal activity of cotton fabric coated with NanoCu* against HCoV-229E coronavirus. A volume of 50 μL of viral inoculum were applied to fabric piece and incubated during 120 min at room temperature. Then, the recovery virus titer was determined by plaque assay (T1). Then, the fabric was washed with distilled water (a) or water +0.5% H_2_O_2_ (b) and virucidal assay was repeated (T2). The process was repeated two times more (T3–T4). *N* = 4 replicates were done, and errors bars correspond to ±SD. Paired Student test analysis were employed for comparing experimental treatments with the control: *: *p* < 0.05. Control (black bars). NanoCu* (gray bars).

Finally, the virucidal efficiency against HCoV-229E coronavirus of the coated cotton fabric after several washing cycles was evaluated (Figure 7). Two different strategies were used: washing with distilled water in each step or directly with a solution of hydrogen peroxide (0.5% from 33% v/v). The full efficiency was conserved in both cases after four cycles, demonstrating that the high stability observed previously is also corroborated in terms of virucidal efficiency. These results therefore validate the potential industrial applicability of this material.

### NanoCu as Additive for Antiviral Paint

**Figure 8:**
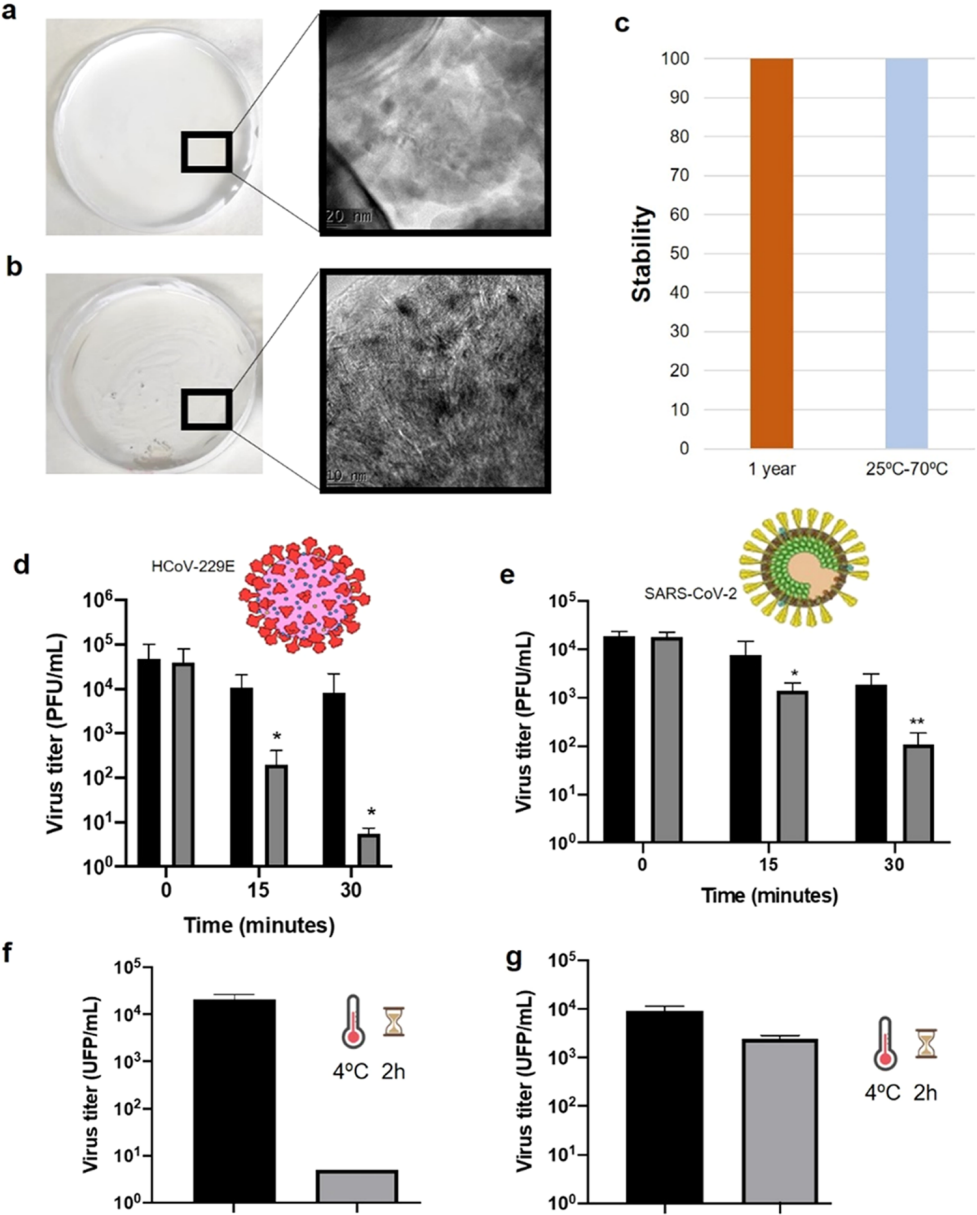
Characterization of the paint samples with NanoCu* solution and the original paint with antivirucidal efficiency. (a) Original paint on the Petri dish surface (HRTEM image). (b) Paint containing 10% NanoCu* on the Petri dish surface (HRTEM image). (c) Stability of NanoCu* in the paint. (d–g) Virucidal effect of NanoCu* against virus. Approximately 10^5^ PFU of HCoV-229E (d) or SARS-CoV-2 (e) were incubated with paint containing 50% NanoCu at different times at 25 °C. Approximately 10^4^ PFU of HCoV-229E (f), SARS-CoV-2 (g) were incubated with paint for 2 h and 4 °C. *N* = 4 replicates were done and errors bars correspond to ±SD. Unpaired Student test analysis was employed for comparing experimental treatments with the control: *: *p* < 0.05. Control (black bars). NanoCu*-paint (gray bars).

Finally, the applicability of NanoCu solution as a paint additive was evaluated. Different concentrations of NanoCu (10, 20, and 50% (v/v) from 5000 ppm solution) were added to a white paint (Figure 8). The paint samples were added to a Petri dish (plastic surface) and then dried (Figure 8a,b). TEM analysis of the paint containing NanoCu demonstrated the presence of the small copper nanoparticles there (Figure 8b). The stability of the material in the paint was also studied, using the catalytic *p*AP assay. This was extremely stable also there, conserving the catalytic efficiency at different *T* from 25 to 70 °C, and also showed full activity over a very long time of incubation, 12-month aging (Figure 8c). Then, the virucidal activity against coronaviruses was tested. For this, the paints (original and treated with NanoCu*) were added to a glass slide and dried (Figure 1). The treated paint showed a high reduction of virus of 4 log 10 (99.99%) after 30 min against HCoV-229E and more than 99% against SARS-CoV-2 at room temperature. Evaluation at 4 °C was also tested and the treated paint also showed virucidal effect at these conditions, 99.99% against HCoV-229E after 120 min. However, the inhibition efficiency decreased against SARS-CoV-2 at 4 °C.

### Sustainability Footprint of NanoCu

Simple and efficient synthesis procedures are mandatory for the final commercialization of a product. NanoCu synthesis is a green and sustainable process carried out in aqueous media at room temperature without the need for special conditions or special equipment, where a cheap enzyme (only 2.9%) is used in the final formation.

The final amount of material added, for example, to complete a fabric of 166 cm^2^ is very low (1–2 mL of 5000 ppm synthesis product, corresponding to 0.0033% of Cu material to total weight (around 3 g)). This means that 1 L of NanoCu would allow the production of 166 m^2^ of coated fabrics.

Furthermore, to assess its efficiency and sustainability footprint, we compared our copper-coated material with one of the most commercially available fabrics that contain copper-containing materials. For example, considering the use of fabrics in face mask commercialization, Cupron company commercializes a fabric product containing more than 600 times more copper content than ours.^23^ Other companies, for example, Kuhn all Copper Mask and Kuhn all Copper Insert, even have products made of 99.95% pure copper mesh. The assessment determined in terms of copper amount, virucidal efficiency, final cost, reusability, stability, and environment sustainability showed that these technologies exhibited medium- or low-level yields mainly because of the moderate stability of the material, high price (more than 10€/unit), no reutilization mechanism, or even very low or negligible virucidal activity at similar copper contents. Our technology presented a high yield considering these parameters with a high stability product, economically potential available fabrics, high virucidal efficacy at low copper content, and very environmentally friendly preparation conditions.

## Conclusions

Antiviral fabrics and paints based on the use of a nanostructured biohybrid material as the coating or additive agent have been fabricated. This represents an ecofriendly and efficient coated system for multiple applications. The simple, sustainable, and scalable method for preparation of the copper nanomaterial makes possible the industrial implementation of the system.

The excellent stability of the nanomaterial in different conditions (high temperature, presence of disinfectants, etc.) has been confirmed even after coating in fabrics or even as a mixture with paints. A high virucidal efficiency has been demonstrated for the coated fabrics or paint against different viruses, in particular SARS-CoV-2. Reusability experiments of the coated materials were performed, demonstrating the self-cleaning capacity of this coating agent, without the necessity of regeneration (conserving the antiviral activity after several cycles).

Therefore, these new coated materials would represent an important element of safety against viruses or other microorganisms, reduce the infections, and avoid transmission. This would be optimal in the case of fabrics; for example, in the fabrication of medical textiles for protection in hospitals, or textiles for use in public transports. In the case of antiviral paints, these would be of interest in public areas such as hospitals, restaurants, etc.

## Supporting Information Available

Supplementary experimental results and data as follows: additional experimental methods, Cu action mechanism against viruses, enzyme structure figure, protocol scheme of NanoCu synthesis, XRD analysis of NanoCu after different experiments or different synthetic batches, SEM analyses of different coated surfaces experiments and virucidal analysis of coated materials (PDF)
