# Supplementary material for: Preparation of Highly Stable and Cost-Efficient Antiviral Materials for Reducing Infections and Avoiding the Transmission of Viruses such as SARS-CoV-2
Source: ACS Appl Mater Interfaces. 2023 Apr 28;15(18):22580–9. doi: 10.1021/acsami.3c03357 (PMC10176473; doi:10.1021/acsami.3c03357)
Supplement: Supplementary file 1 — am3c03357_si_001.pdf [file am3c03357_si_001.pdf]

Supporting Information

Preparation of High Stable and Cost-efficient Antiviral Materials for Reducing Infections and Avoiding the Transmission of Viruses such as SARS-CoV-2.

Noelia Losada-Garcia[1], Angela Vazquez-Calvo[2], Antonio Alcami[2] and Jose M. Palomo[1]*

[1]Instituto de Catálisis y Petroleoquímica (ICP), CSIC, C/ Marie Curie 2. 28049 Madrid, Spain, e-mail: josempalomo@icp.csic.es

[2]Centro de Biología Molecular Severo Ochoa, Consejo Superior de Investigaciones Científicas (CSIC)-Universidad Autónoma de Madrid (UAM), 28049, Madrid, Spain

Additional Methods:

Stability of NanoCu under different conditions

Stability against contact with different disinfectants

To evaluate the effect of disinfectants on the stability of NanoCu previously adhered to a certain surface, samples of 0.25 g of solid were prepared with 800 µL of ethanol: water 50:50, each of which was added to a Petri dish. Once air dried (1-3 h), 600 µL of each disinfectant was sprayed. The disinfectants used were 3% hydrogen peroxide (obtained by diluting a 33% hydrogen peroxide solution), 0.1% bleach (obtained by diluting commercial bleach (40 g sodium hypochlorite/L) in water), 70% and 96% ethanol, 70% isopropanol and 1.75% hexadecyltrimethylammonium bromide (CTAB). After air drying

in a Petri dish, the solid was recovered. To verify that there was no change in the chemical species of the sample, they were analysed by X-ray diffraction (XRD).

Stability against incubation in the presence of additives described as disinfectants

To assess the stability of NanoCu mixed directly with the disinfectant, 5 mg of the material was incubated in 7.5 mL of a solution (0.66 mg/mL) of 0.1% bleach, 70% isopropanol, or CTAB 1.75 %, evaluating its stability after 7 days of incubation. After this incubation time, the solids did not undergo any type of change in colour or morphology, the copper species (demonstrated by X-rays) and the amount of copper (quantitative analysis) being completely preserved. Furthermore, it was confirmed that the catalytic efficiency of the nanoparticles in NanoCu was preserved at 100% after the incubation time.

Stability under high temperature

Another test performed to evaluate the stability of NanoCu was incubation at high temperature. In this case, the stability of the chemical structure was evaluated by exposing the material for 1 h at 100ºC in water and air, and at 150ºC in an oil bath. The solids after treatment were evaluated by X-ray diffraction. There were no changes in the chemical species of NanoCu, and therefore the coating is stable at high temperature.

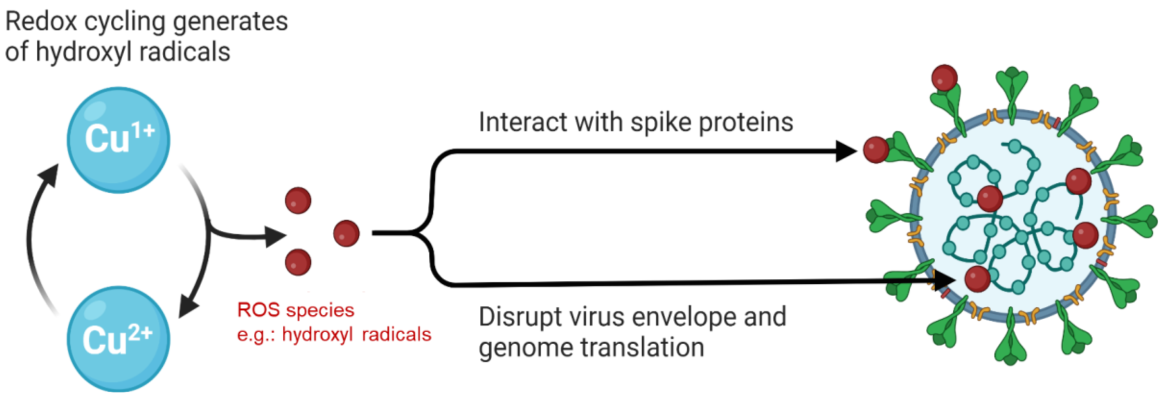

Figure S1. Proposal Cu-action mechanism against viruses.

A B

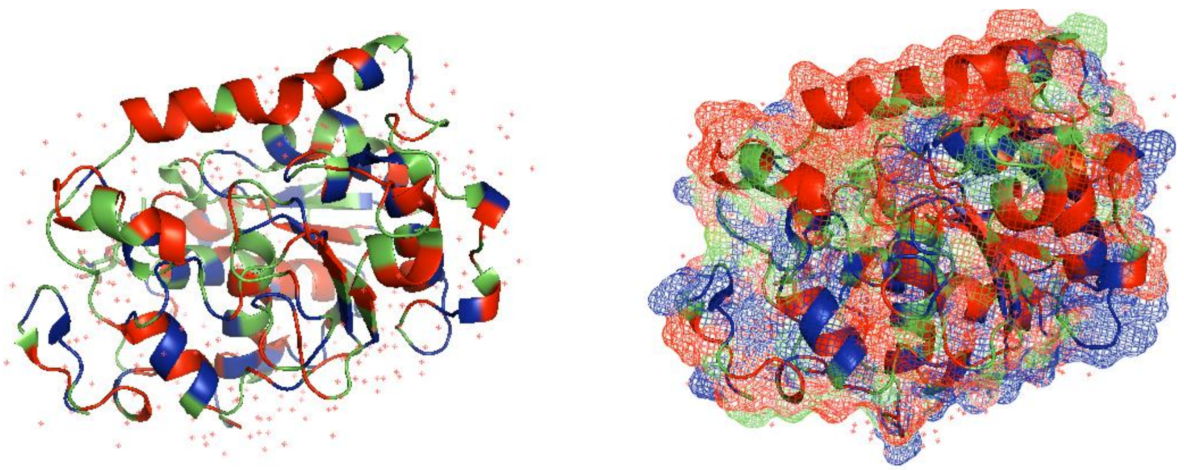

Figure S2. Three-dimensional structure of CALB TyrAB. A) Cartoon backbone representation. B) Cartoon backbone with mesh representation. Hydrophobic residues (red), Hydrophilic residues (blue).Structure was obtained from PDB data bank with following code: 1TCA. Figure was drawn using Pymol program.

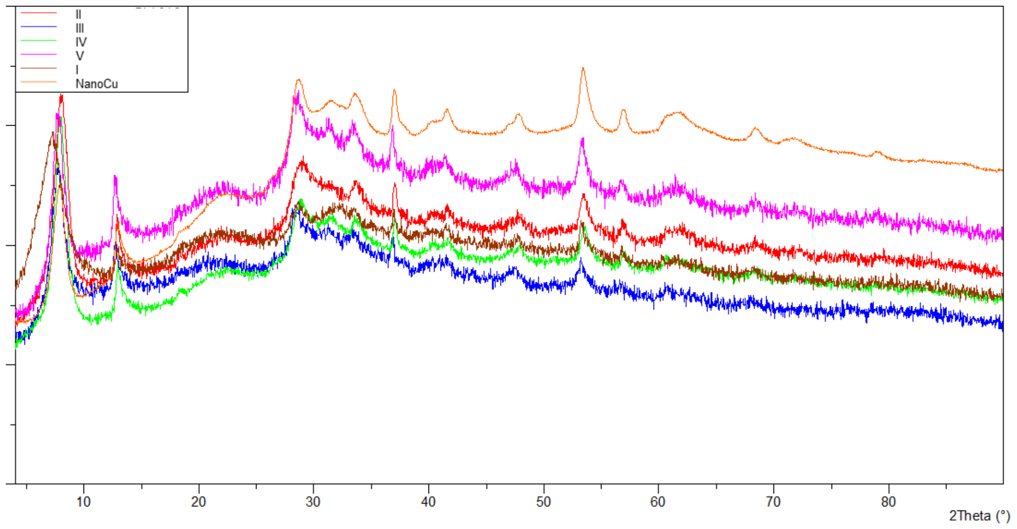

Figure S3. X-ray diffraction spectra (XRD) of the solids after disinfectant application compared to the untreated solid. **I)** Multipurpose disinfectant (0.6% didecyldimethylammonium chloride); **II)** 3% H_2O_2; **III)** 0.1% bleach; **IV)** 70% ethanol; **V)** 96% ethanol, **VI)** 70% isopropanol, **VII)** 1.75% CTAB

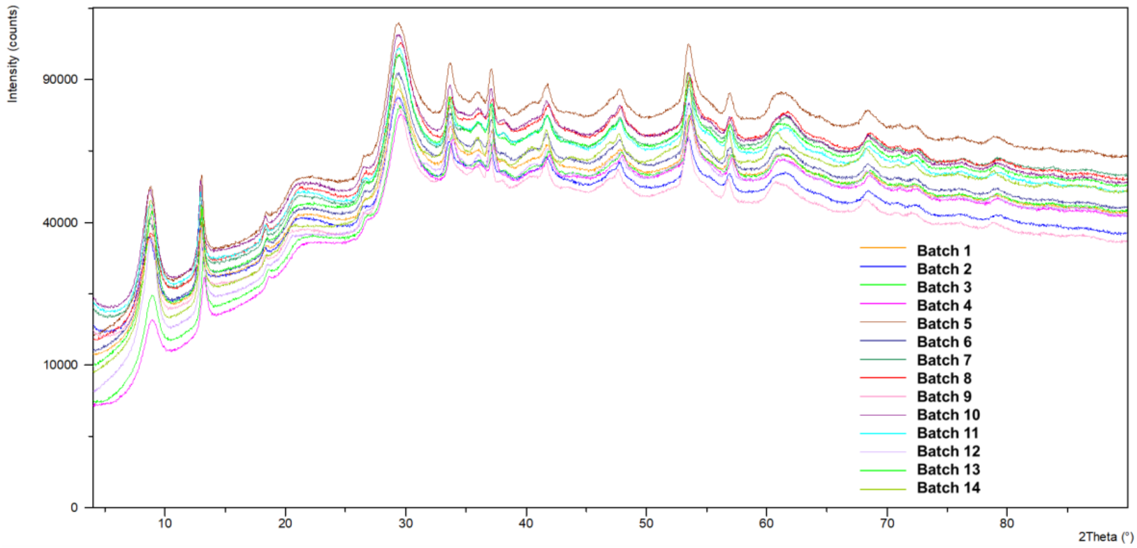

Figure S4. XRD spectra of NanoCu with different batch of production

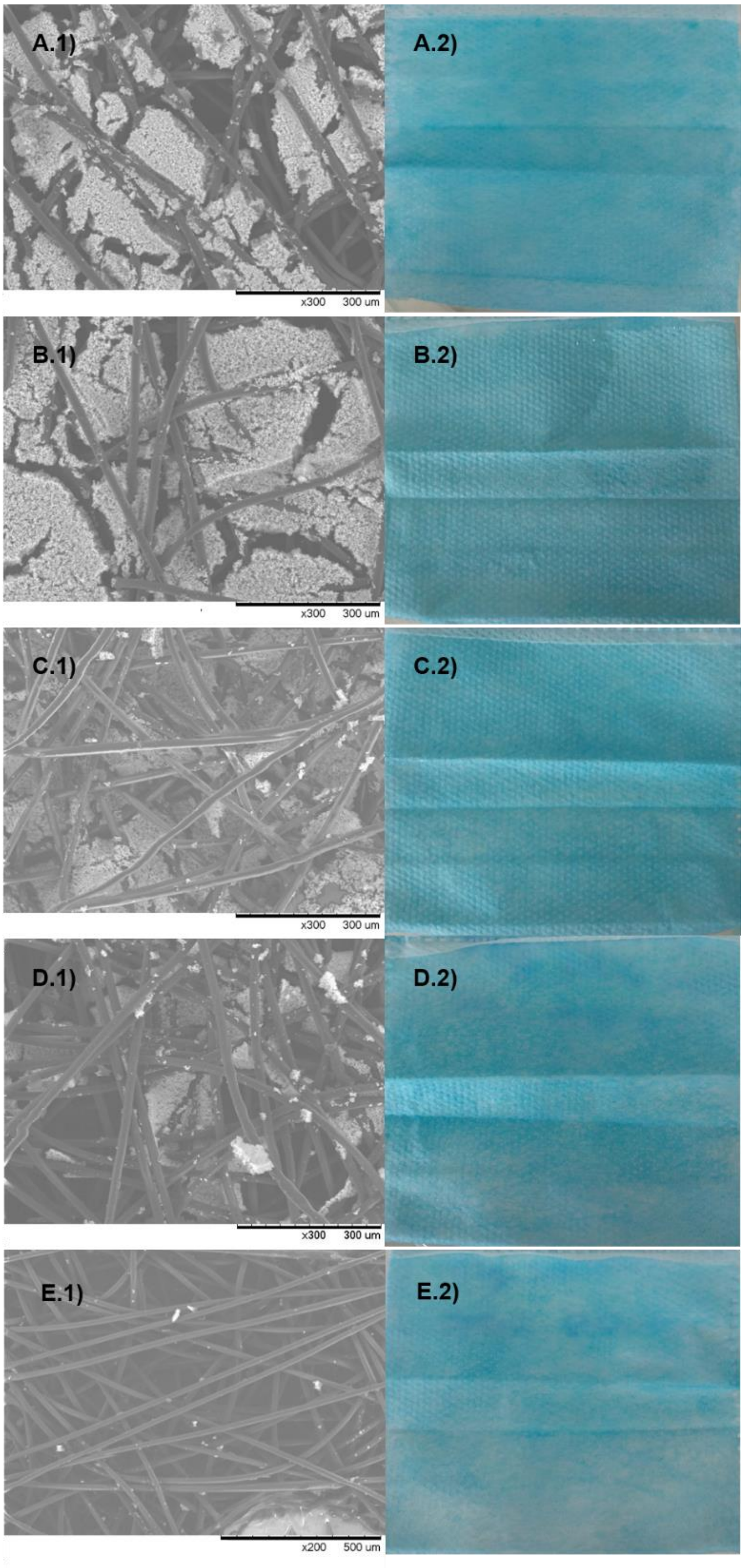

Figure S5. 1) SEM microscopy images of the structure of the surgical mask with the coating with NanoCu in ethanol:water 50:50; **2)** Image of the actual mask. **A)** 1000ppm; **B)** 750ppm; **C)** 500ppm; **D)** 250ppm; **E)** 125ppm. All samples have been made in $45cm^2$.

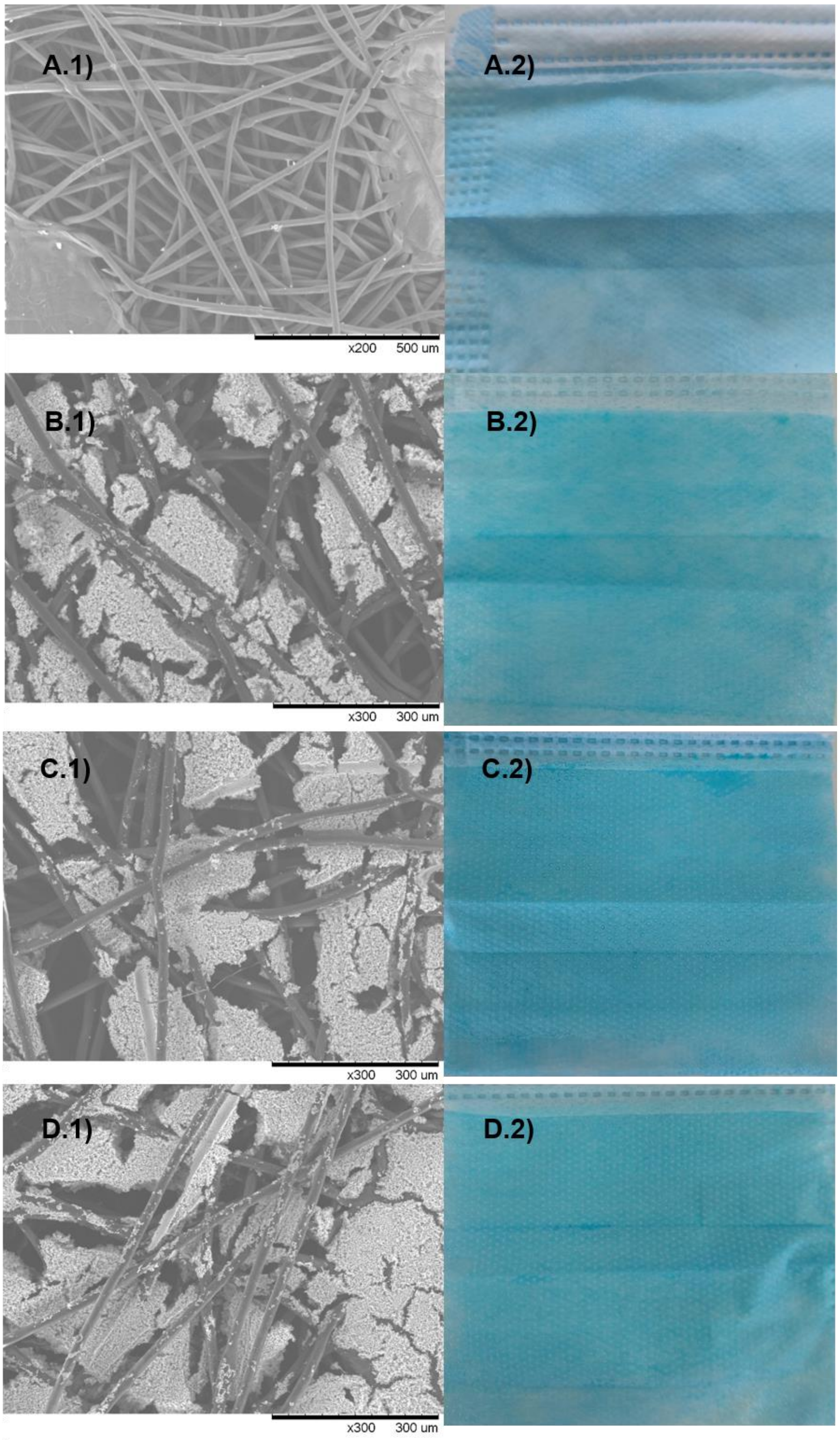

Figure S6. **1)** SEM microscopy images of the surgical mask structure. **2)** Image of the actual mask. **A)** Untreated surgical mask; **B)** Mask with NanoCu in Ethanol:water 50:50; **C)** Mask with NanoCu in Ethanol:water 70:30; **D)** Mask with NanoCu in Ethanol:water 80:20. All samples have been made in 45cm^2 with 1000ppm of NanoCu.

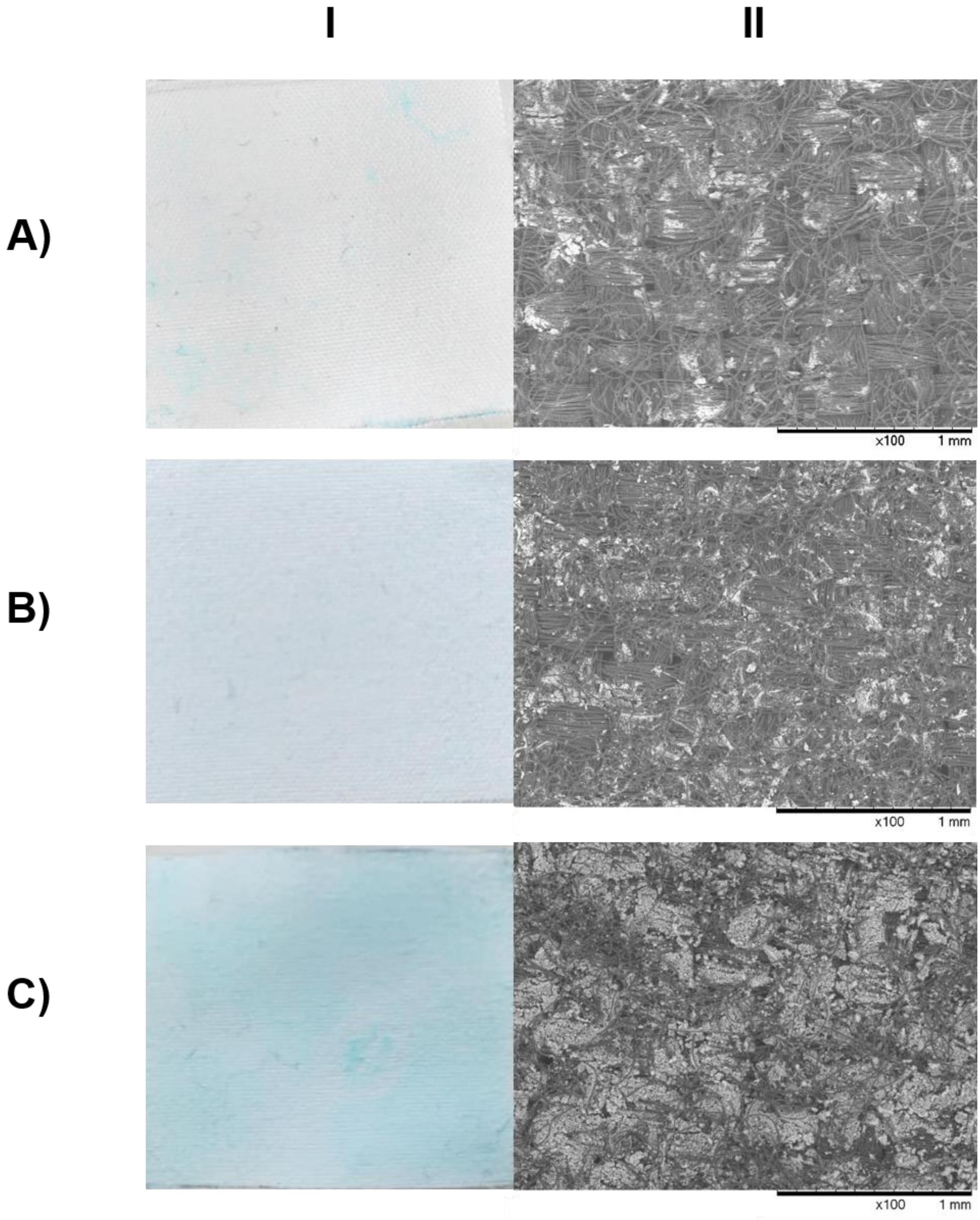

Figure S7. White polystyrene fabric tests: **1)** Image of the actual fabric; **2)** SEM microscopy images of the structure of the polystyrene fabric with the coating NanoCu in ethanol:water 50:50. **A)** 125ppm; **B)** 250ppm. **C)** 350ppm; All samples have been made in 10.5cm^2.

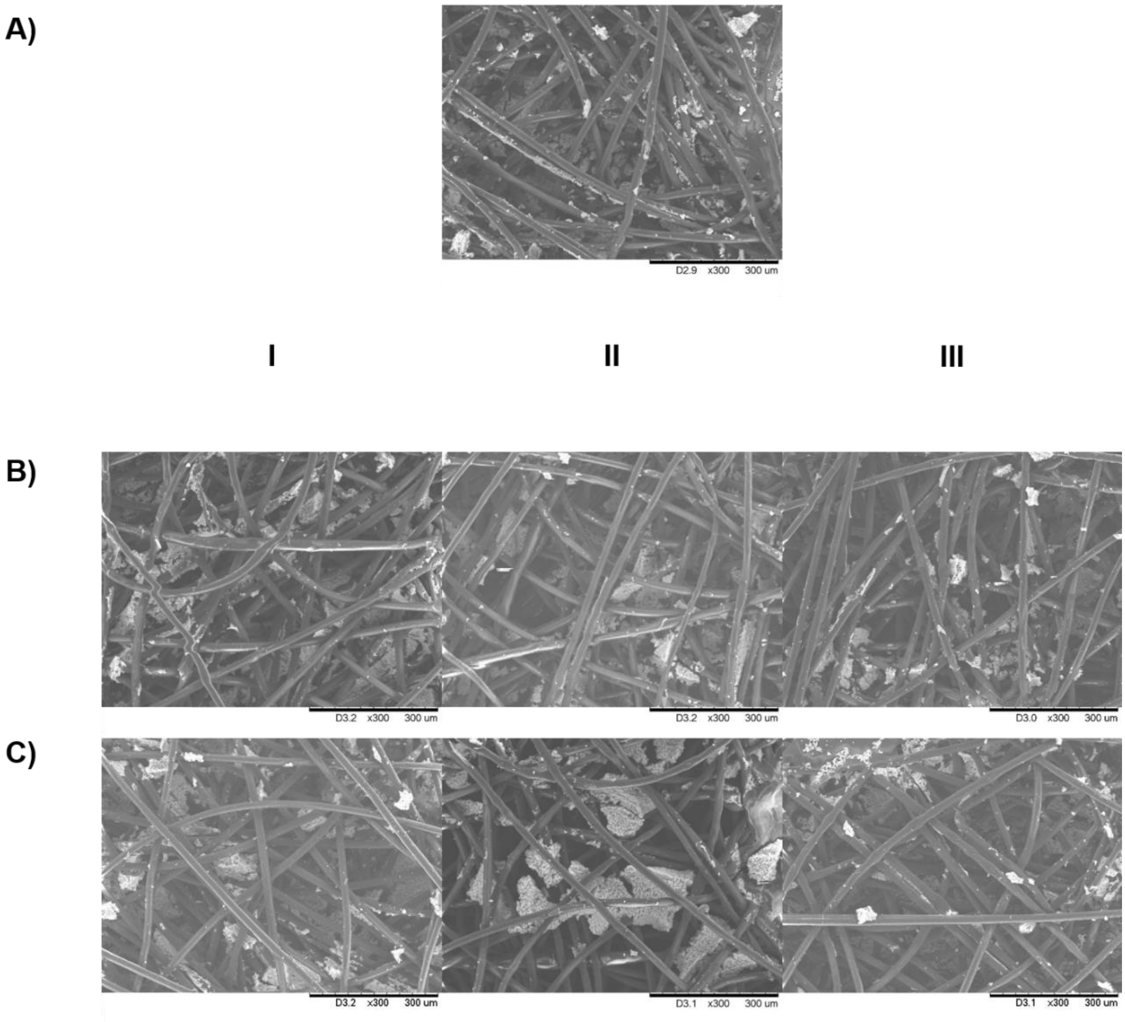

Figure S8. SEM microscopy images of the NanoCu-coated polypropylene fabric against washes (125ppm of NanoCu in ethanol:water 50:50). **A)** Untreated sample; **B)** Samples at 40ºC; **C)** Samples at 60ºC. **I)** 30 min; **II)** 60 min; **III)** 120 min.

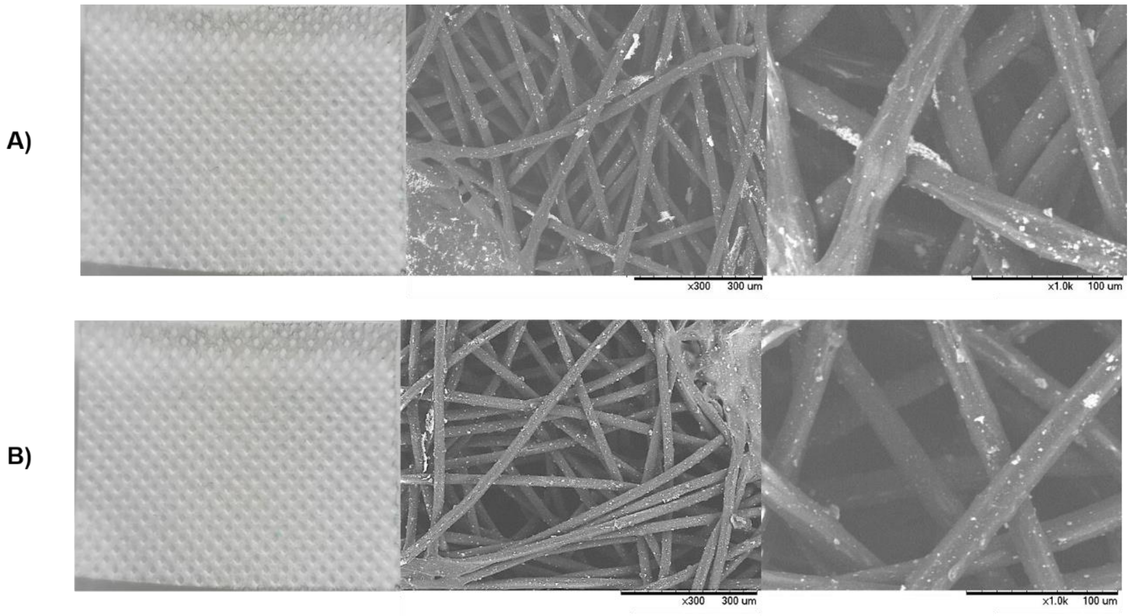

Figure S9. **A)** SEM images of the filter polystyrene fabric with the immersion method without agitation at a concentration of 625 ppm for 1 min; **B)** SEM images of the fabric with the immersion method with agitation at a concentration of 1250ppm for 2 min. All samples have been made in 10.5cm^2.

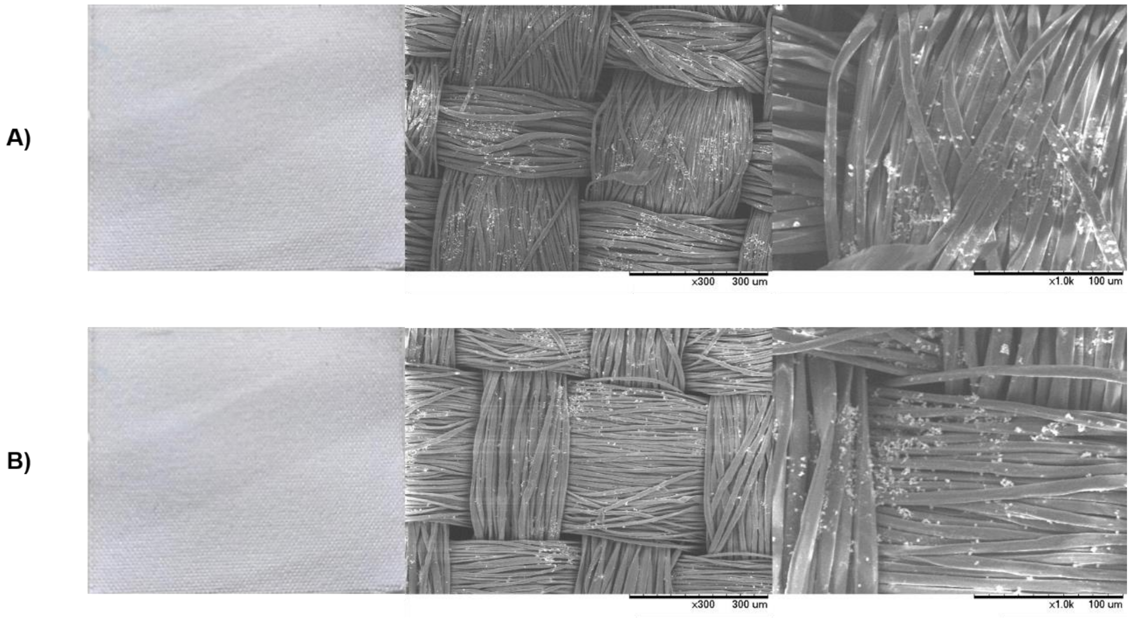

Figure S10. A) SEM images of the white polyester fabric with the immersion method without agitation at a concentration of 625 ppm for 1 min; **B)** SEM images of the fabric with the immersion method with agitation at a concentration of 1250ppm for 2 min. All samples have been made in 10.5cm^2.

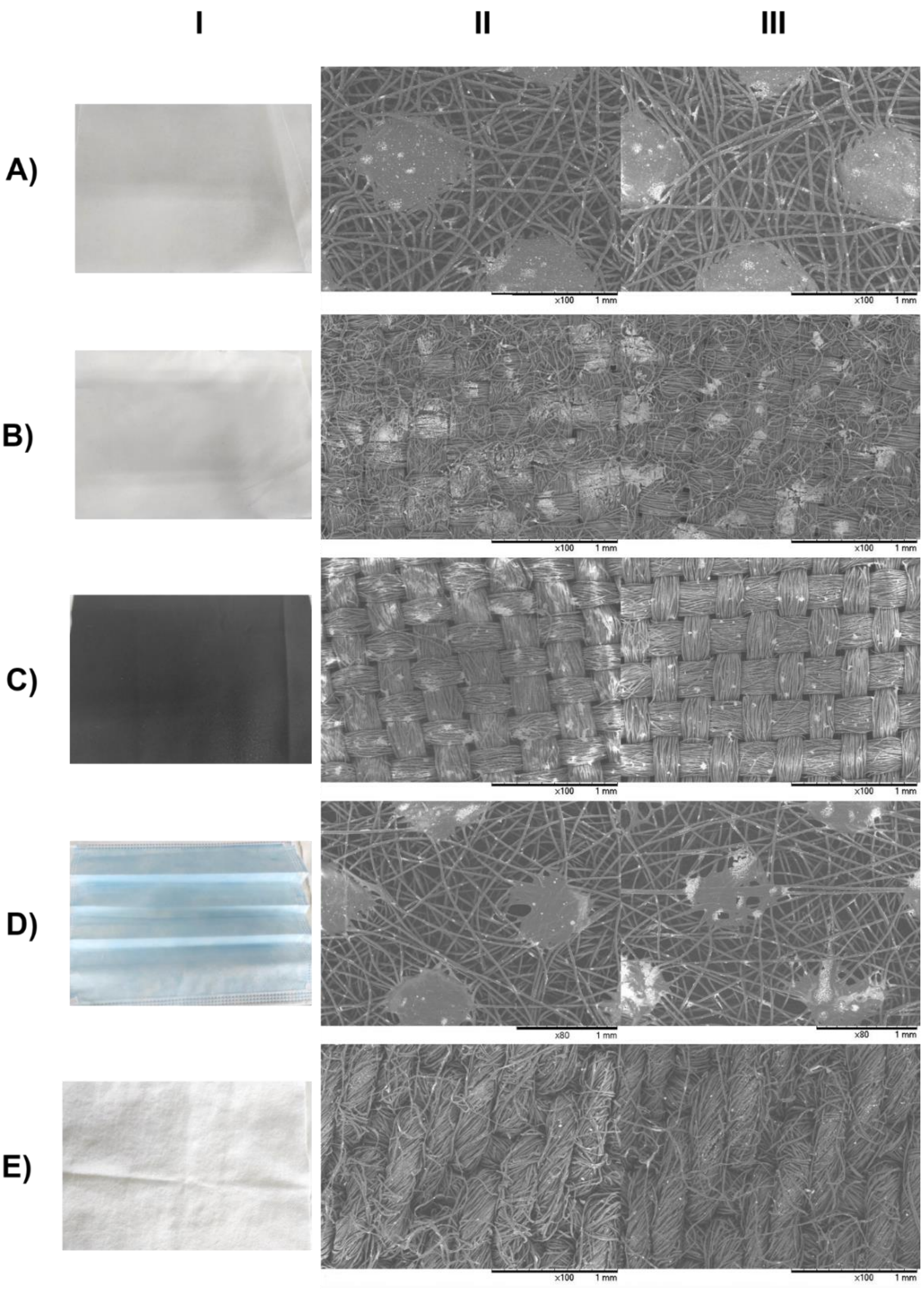

Figure S11. Application of NanoCu by spraying in different positions. **I.** Real piece of mask and fabrics; **II.** 9 sprays at 5 cm from the paper horizontally (NanoCu concentration -1250ppm); **III.** 15 sprays at 5-10 cm from the paper vertically (NanoCu concentration - 1250ppm). **A)** 100% cotton fabric; **B)** White polystyrene fabric; **C)** Filter polystyrene fabric; **D)** polypropylene fabric.

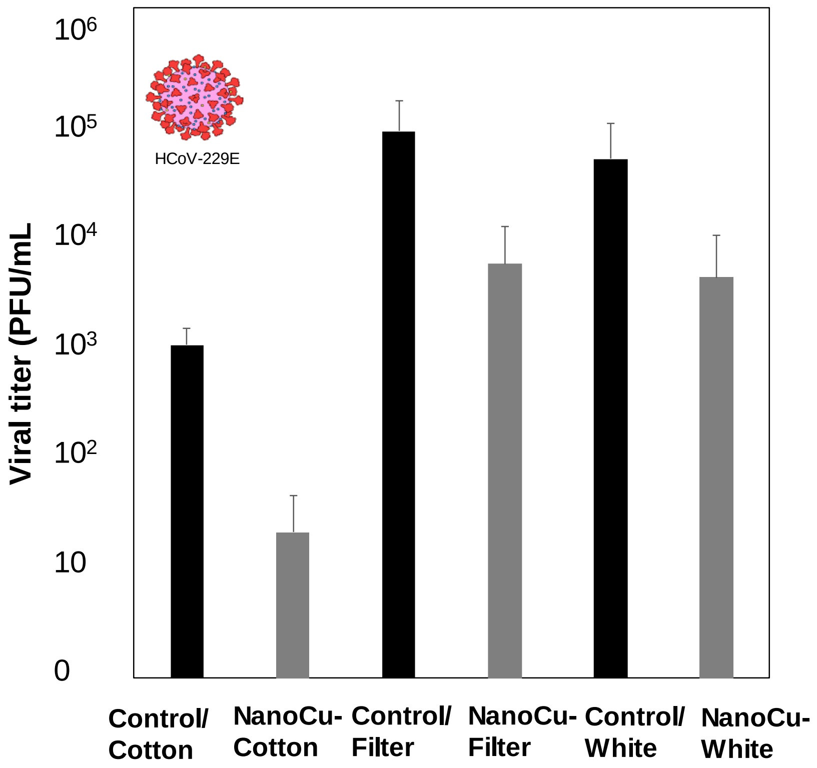

Figure S12. Determination of virucidal activity of different fabrics with NanoCu incorporated against HCoV-229E coronavirus. A volume of 50 µL of viral inoculum containing approximately 10^5 PFU were applied to different textiles and incubated for 120 minutes at room temperature. Then, the recovery virus titer was determined by plaque assay. N = 4 replicates were done, and errors bars correspond to ±SD. Control without fabric or uncoated-fabric (black bars), NanoCu-coated fabric (grey bars).

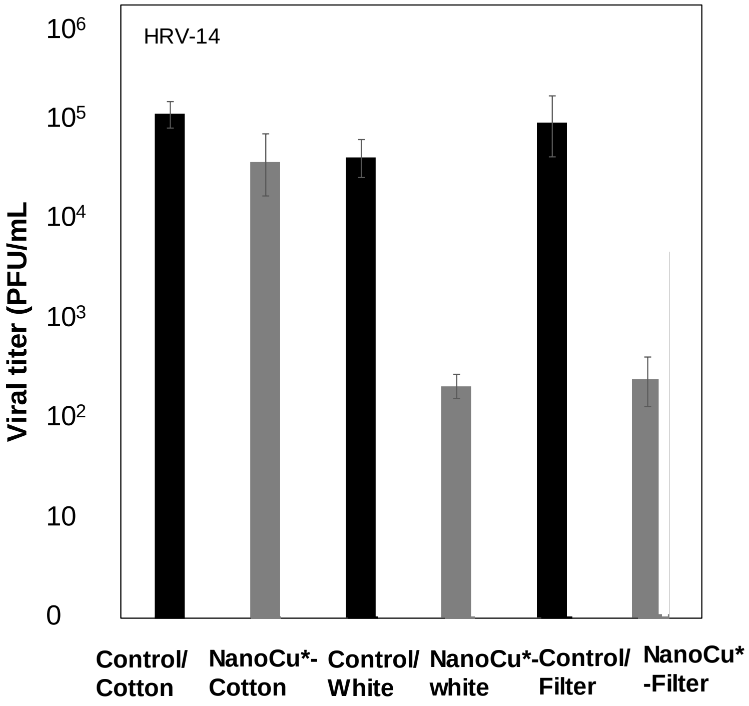

Figure S13. Determination of virucidal activity of different fabrics with NanoCu* incorporated against coronavirus. A volume of 50 µL of viral inoculum containing approximately 10^5 PFU of HRV-14 virus were applied to different textiles and incubated for 120 minutes at room temperature. Then, the recovery virus titer was determined by plaque assay. N = 4 replicates were done, and errors bars correspond to ±SD. Paired Student test analysis were employed for comparing experimental treatments with the control: *: $p < 0.05$; **: $p < 0.01$; ***: $p < 0.005$; ****: $p < 0.001$. Control without fabric or uncoated-fabric (black bars), NanoCu*-coated fabric.
